# Negotiating pain: the joint construction of a child's bodily sensation

**DOI:** 10.1111/1467-9566.12207

**Published:** 2015-03-10

**Authors:** Laura Jenkins

**Affiliations:** University of Sheffield

**Keywords:** conversation analysis, children, pain, participation, agency

## Abstract

Traditional theories of socialisation, in which the child was viewed as a passive subject of external influences, are increasingly being rejected in favour of a new sociology of childhood which frames the child as a social actor. This article demonstrates the way in which conversation analysis can reveal children's agency in the micro-detail of naturally occurring episodes in which children express bodily sensations and pain in everyday life. Based on 71 video-recordings of mealtimes with five families, each with two children under 10 years old, the analysis focuses on the components of children's expressions of bodily sensation (including pain), the character of parents’ responses and the nature of the subsequent talk. The findings provide further evidence that children are social actors, active in constructing, accepting and resisting the nature of their physical experience and pain. A conversation analysis of ordinary family talk facilitates a description of how a child's agency is built, maintained or resisted through the interactional practices participants employ to display knowledge.

## Introduction

As assumptions about the nature of childhood have developed, the view that the child is merely subject to social forces is increasingly being rejected in favour of a new sociology of childhood that frames the child as a social actor (Moran-Ellis 2010). This emergent paradigm, which stands in contrast to a traditional framework that positioned children as passive victims of external influences (Alanen 1988), instead understands childhood as a social construction; a negotiated set of social relationships in the early years of human life (Prout and James 1997). Alongside this, the concept of intergenerational relations has been highlighted as being useful in understanding children's experiences of childhood, particularly the ways in which children's lives are structured through their relationships with adults (Mayall 2001a).

Parallel developments are evident in the way listening to children's voices is increasingly promoted in both policy (Department of Health 1989, Lansdown 2001, UN 1989) and social science method (Prout and James 1997), with a move towards research that explores children's experiences in their own terms (Prout 2002). Constructing children as agents in their own lives is operationalised in terms of granting them the status of participants and constructors, specifically in social science research (Prout and James 1997). Utilising participatory methods has been advocated as a means by which to address the inherent power relations involved in the research process (Moran-Ellis 2010, Prout 2002), in addition to interactionist analysis that subjects aspects of everyday life to detailed and critical reflection (Prout and James 1997).

Interactionist methodologies address concerns about how children interact with each other and with adults, and how they shape as well as are shaped by the various locales of their lives (Prout 2002). Conversation analysis (CA), in particular, has proven to be a useful way of examining how talk is used to manage social relationships and construct identities (Drew 2005), seeking to describe in extensive detail the structures and patterns that enable us to understand how speakers do what they do and understand what others do (Schegloff 2005).

CA has been applied to talk about pain, but in institutional settings. This has proven to be a valuable means by which to describe how the revelation of pain emerges in the sequential progression of certain medical actions and activities (Heath 1989) and to highlight children's agency in terms of how children are able to solicit parental assistance in ways that underline the role of the child as the primary informant (Clemente 2009). CA's systematic approach to examining the fine detail of interaction represents a unique tool for exploring the emerging sociology of childhood's focus on children's agency and social relationships (Prout and James 1997).

In contrast to interactionist methodologies that seek to explore intergenerational relations by asking children to describe their experiences of childhood (for example, Mayall 2001b), the current study used CA to analyse video-recordings of talk between children and their parents. It investigates the negotiation of the status of symptoms of illness and pain as expressed by children to their parents in everyday settings. It considers children's agency in their health in terms of how the nature, severity and authenticity of their sensations (including pain) are socially constructed and managed on a turn-by-turn basis, shaped by contributions from both children and parents. It investigates how children's rights to report on their own experience are produced, accepted or resisted by children and parents, evidencing the social construction of childhood and children's agency in the micro-detail of concrete instances of naturally occurring talk. These findings are discussed in terms of their implications for how authority and rights to access an experience or knowledge about illness are constructed in the claims embedded in talk.

## Aims

The objectives of this study are to present a systematic and detailed examination of video-recorded episodes in which children express bodily sensations, including pain, in everyday family life, and to reveal the way in which the characterisation of the bodily sensations are shaped by contributions from both children and parents. The analysis investigates intergenerational relations by specifically seeking to (i) examine how children exert agency and how the nature of their sensation is formulated in their expressions of bodily experience; (ii) reflect on how a parent's response reworks the nature of the child's experience; and (iii) describe how a child's right to report on their own sensation is negotiated in the subsequent interaction.

## Method

The analysis in this article is based on a corpus of video-recordings of family mealtimes. Full ethical clearance was granted by Loughborough University's ethical committee. The participants were recruited via voluntary support groups for families with children with chronic health concerns, on the basis that talk about a child's health and their body may be more prevalent in such families. Organisations that focused on diabetes, heart conditions and allergy distributed information about the project to approximately 600 potentially eligible families in the UK. The adults and children of three families responded and signed consent forms. They were given a video camera and asked to film between 10 and 15 meals. When to film was at the participants’ discretion (with the exception of mealtimes in which guests attended who had not given informed consent to be recorded), and even after having filmed a meal they were able to delete any recordings they did not wish to submit to the study. The participants were told that they had the right to withdraw their data at any time, including after the point at which they had returned the camera and all the recordings they had made.

Recordings from a further two families were accessed through an archive of recordings kept by the Discourse and Rhetoric Group at Loughborough University.^1^ These families were recruited on the basis that they ate their meals together, and in this study these particular families provide a broader data set and offered a potential comparison with the families in which particular health issues were of concern. In total, the five families recorded 71 mealtimes, totalling 32 hours of data. This represented an amount of data that was both manageable, in terms of being systematically searched, and sufficient, in terms of yielding the collection of interactional phenomena forming the focus of this study. The participants were all white and of middle socioeconomic status. Each family included a heterosexual married couple and two children under 10 years old (see Table1 for more details). All names have been changed.

**Table 1:** Participants’ details

*Family*	*Number of meals filmed*	*Age of children*	*Recruitment Source*	*Health conditions*
Edwards	14	Lanie (4 y) Finley (15 m)	Support group	Lanie has a congenital heart condition
Hawkins	14	Jack (9 y) Charlie (5 y)	Support group	Jack has type I diabetes
Jephcott	17	Haydn (6 y) Isabelle (4 y)	Support group	Haydn and Isabelle have food allergy and intolerance issues
Amberton	13	Emily (7 y) Jessica (4 y)	DARG Archives	None recorded
Crouch	13	Katherine (5 y) Anna (3 y)	DARG Archives	None recorded

m, month; y, year

Like Clemente's (2009) study and other CA work in medical sociology (for example, Parry 2004, Pilnick and Zayts 2012), this article provides a data-driven analysis of talk-in-interaction. CA sets out to understand the elementary properties of social action (Schegloff 1992), in order to understand how talk is used to manage social relationships and construct, establish, reproduce and negotiate identities (Drew 2005) (For a more in-depth description of CA's analytic assumptions and method see Drew 2005, Heritage 1984, Schegloff 2007). The selected extracts were transcribed using the conventions developed by Gail Jefferson and supplemented by Hepburn in relation to distress and upset (Hepburn 2004, Jefferson 2004) (Table2).The analysis in this article focuses on a collection of sequences in which children initiate an expression of bodily sensation, including pain. It describes the components of children's expressions, the character of the parents’ responses and the nature of the subsequent conversation, revealing the way in which children exert agency and participate in talk about their bodies.

**Table 2:** Transcription conventions

[ ]	Square brackets mark the start and end of overlapping speech.
↑	Vertical arrows precede marked pitch movement.
Underlining	Indicates emphasis.
CAPITALS	Mark speech that is hearably louder than surrounding speech. This is beyond the increase in volume that comes as a by product of emphasis.
^°^I don't know^°^	‘Degree’ signs enclose hearably quieter speech.
#My tummy hurts.#	Hash signs denote creaky delivery
(0.4)	Numbers in round brackets measure pauses in seconds (in this case, 4 tenths of a second).
(.)	A micropause, hearable but too short to measure.
((grabs head))	Additional comments from the transcriber, e.g. about embodied actions.
my tu:mmy h:ur:ts	Colons show degrees of elongation of the prior sound.
.hhh	Inspiration (in-breaths).
Yeh,	‘Continuation’ marker, weak rising intonation.
what sorry?	Question marks signal stronger, ‘questioning’ intonation, irrespective of grammar.
Right.	Full stops mark falling, stopping intonation (‘final contour’), irrespective of grammar.
I'm probably s-	hyphens mark a cut-off of the preceding sound.
>I know<	‘greater than’ and ‘lesser than’ signs enclose speeded-up talk.
solid.= =We had	‘Equals’ signs mark the immediate ‘latching’ of successive talk.

For more detail see Jefferson (2004) and Hepburn (2004).

## Findings

The corpus of mealtimes contained various talk relating to health, pain and the body, such as discussions about a friend being treated in the burns unit, talk about a child's medication, parents' reports of pain or parents initiating enquiries into a child's wellbeing. This study focused specifically on sequences in which children initiated expressions of bodily sensation, on the basis that they were more prevalent in the data and represented a distinct set of phenomena for elucidating commonalities in how they were formulated and delivered. In total, 33 expressions of bodily sensation which initiated new sequences were produced by children. These were found in 20 mealtimes across all five families. A careful descriptive account of these pain expressions is provided elsewhere (Jenkins 2013).

In this corpus episodes containing expressions of physical experience rarely contain what is described in CA as an adjacency pair. This is a simple unit of talk containing a turn from one speaker, followed by a second turn (a response) from another speaker (Schegloff 2007). Instead, they often involve extended sequences in larger stretches of talk during which the nature of the sensation is negotiated. Sequence organisation is an orderly turn-by-turn process that provides slots where adults or children can resist or accept the diagnostic work of the other speaker, display understanding, repair their own or another speaker's talk of the previous turn and negotiate the nature of the bodily sensation and the response. Extensive stretches of talk can be built as a way of dealing with difficulties in the talk, such as negotiating the nature of the child's experience (Schegloff 2007).

In this analysis I show how the character of the sensation is initially formulated within the child's report and embodied in these reports are claims of unmediated access to their experience, and the right to report on these sensations. In their responses parents also make claims about the nature of the child's experience, its severity and its authenticity, and potential remedies. The analysis that follows describes the strategies by which children can accept or resist the claims embedded in these responses.

Example 1: Child accepts adult's non-serious interpretation

In this first example, Lanie's report that her tummy hurts is responded to by Dad, who claims that he doesn't think it hurts and suggests an alternative, non-serious interpretation of the sensation:

Extract 1: Edwards 5: 16.58–17.04 You're quite full

**Table:** 

67 Lan**:**	Oh my tu:mmy h:ur:ts.
68 Dad**:**	I ↑don't think it hurts Lanie. It's probly just
69	cause you're quite full.
70	(0.5)
71 Lan**:**	Yeah.

In line 67 Lanie reports that her tummy hurts, producing a lexical formulation that identifies the location (‘tummy’) and the nature of the sensation as hurting. As with many of the lexical formulations in this collection, this formulation is characteristic of an announcement; an assertion relating to a current, speaker-specific event (Schegloff 1995). Connected with this it contains an indication that the reported condition is current such that the recipient would not be aware of it (Maynard 1997, Schegloff 1995). The turn is prefaced with an ‘oh,’ which signals a change of state, displaying the newness of this feeling (Heritage 1984). By announcing the condition as news, and claiming ownership of the sensation, the child treats the adult as unknowing, that is, as not having access to the child's experience.

Dad produces a second pair part with no delay, asserting that he doesn't think her tummy hurts, and provides the explanation ‘It's probly just cause you're quite full’ (line 68). Dad resists Lanie's characterisation of the sensation as ‘hurting,’ and formulates an alternative explanation which minimises the seriousness of the experience, invoking the notion that it is normal and ordinary; fullness rather than pain. It also does so with an orientation to the potentially disaligning nature of the explanation, employing the terms ‘think’ and ‘probly’, which soften the strength with which Dad asserts this knowledge. In this way the epistemic claim (that is, the extent to which Dad asserts rights to knowledge about the diagnostic explanation underpinning Lanie's experience) is weakened.

After a short delay Lanie responds in third position on line 71 with a receipt that aligns with Dad's explanation. This token ‘Yeah’ provides an acceptance of the less serious interpretation, making no issue with Dad's epistemic claims to access her experience, but which nonetheless positions Lanie as able to confirm or refute the assessment. It is a minimal post-expansion, designed to close the sequence (Schegloff 2007).

This extract demonstrates the way in which the nature of the sensation, produced initially by the child, is reworked by the response, and the alternative formulation is accepted by the child in third position. The child demonstrates rights to report on their bodily sensations, and to accept or deny reworked interpretations of the sensation. An explicit acceptance of the parent's interpretation of their sensation is rare in this data corpus. Elsewhere, the sequential space following an adult's response is used by the child to resist an adult's formulation of the nature of the experience, as the next example shows:

Example 2: Child resists adult's formulation that the expression is exaggerated

In the next extract, Dad has offered Jack second helpings of dinner. As we join the talk, Dad is scraping the spoon in the saucepan, causing a high-pitched sound.^2^ As Jack is accepting the offer he puts his hands on his ears and before completing his turn produces a cry of pain:

Extract 2: Hawkins 11:10.05–12.29 Bit over the top

**Table:** 

130 Jack:%	Okay [I'll have some]
131%	(5) [((Jack puts hands on ears))]
132 Dad:	chicken.=
133 Jack:	=AH: .hh A:H:
134	(1.5)
135 Mum:	He doesn't li:ke that sound. ↑You
136	do'are a bit over the top [ ( ) ]
137 Jack:	[But it ] Doe:s
138	give me like that.
139 Mum:	>I know< but it's a bit th:eatrical isn't
140	it.

In line 133 ‘AH: .hh A:H:’ provide no detail as to the nature of the pain, although the embodied actions in response to scraping sounds from spooning food from the saucepan provide sources by which the other participants can infer the source. After a 1.5 second gap, Mum asserts ‘He doesn't li:ke that sound.’ which, according to the person reference seems to address Dad regarding Jack. She formulates Jack's experience in terms of a preference rather than sensations or unpleasant feelings. She then changes to the first person, addressing Jack in the next unit of talk, known in CA as a turn construction unit or TCU (Sacks *et al*. 1974) saying ‘↑You do'are a bit over the top.’ The expression of pain is packaged as disproportionate to the level of pain actually experienced, undermining the legitimacy of his expression. The present-tense formulation offers the description as an instance of a more generalised pattern, and in this way produces ‘being over the top’ as a dispositional character of the speaker (Edwards 1995).

The end of Mum's turn on line 135–136 is inaudible, and Jack, in third position, produces a turn in overlap that seems defensive, either of his experience or his actions, with the contrastive term ‘But’ which is produced in turn-initial (at the start of the turn) and places an emphasis on ‘does’ when he says ‘it Doe:s give me like that.’ Mum goes on to assert ‘>I know< but it's a bit th:eatrical isn't it’. The tag question ‘isn't it’ produces the assertion as if Jack should be in agreement. In this way Mum orients to the notion that other speakers have privileged access to their own experiences, with specific rights to narrate them (Sacks 1984) and invites Jack to agree.

In Extract 2 it is possible to see how claims to knowledge about Jack's experience are negotiated. Participants in interaction have been shown to display sensitivity to what they have rights to know, how they know it, and whether they have rights to say it, and they constantly manage their and others’ rights to describe or evaluate states of affairs (for a more detailed discussion of epistemics see Heritage 2012; Raymond and Heritage 2006). Mum's initial response displayed an understanding of Jack's report, reworking the expression in terms of its authenticity. As in example one, Jack uses the third position to assert his rights to confirm or deny Mum's claims, in this case resisting the embodied assumptions about both the nature of this expression, and Jack's ‘theatrical’ disposition more generally.

Example 3: Child resists parent's serious diagnostic explanation

Whereas Extract 2 provides an example of the child resisting a response which undermines the legitimacy of the bodily sensation, in Extract 3 the child resists a diagnostic explanation that pathologises the reported sensation. This mealtime is breakfast and the extract begins 15 minutes into the meal, following a lapse in talk, when Lanie asserts that her tummy hurts, a sensation that Mum proposes will be alleviated by drinking water as the pain is probably caused by constipation:

Extract 3: Edwards 5:15.50–16.50 Probably a bit constipated

**Table:** 

05	(10.7)
06 Lan:	[ #My tu:mmy ] [ hur:ts:.# ]
07	[((Lanie leans heads))] [((Lanie puts hand on tummy))]
08	[ (1.9) ]
09	[((Lanie puts hand in lap))]
10 Mum:	Mm thi:nk (1.6) need to have full day:, of
11	getting you drinking cause I think you're probly
12	a bit (1.2) co:nstipay::ted,
13	(0.9)
14 Lan:	I ar:m't,
15 Mum:	Mm:, either that or you've got a tummy bug.
16	(2.5)
17 Lan:	U:h
18	(1.0)
19 Lan:	I've got no:thing you two,

On line 6 Lanie produces an assertion that her tummy hurts, claiming unmediated access to this sensation, conveying information related to the nature of the pain and her experience of upset without marking her epistemic rights to remark on the matter (Heritage and Raymond 2005). Lanie's formulation is followed by a gap of 1.9 seconds. While Mum (or Dad) may be chewing at this point, they both display the ability to talk through a mouthful elsewhere, and this gap signals some sort of delay, indicating a dispreferred response (Pomerantz 1984).

Mum's turn beginning in line 10 is formulated as an assertion that proposes a remedy (drinking) and an explanation (constipation). In this way the response embodies an understanding of Lanie's reported sensation as genuine and legitimate and suggests a potential course of action for remediating it.

Mum infers that Lanie's pain is a serious (though acute and solvable) condition. However it is produced in a manner that mediates the rights Mum has to assert a diagnostic explanation. The turn begins with ‘Mm thi:nk.’ which presents the turn as a perspective display. Like subjective-side assessments (Wiggins and Potter 2003), this diagnostic explanation is marked as the speaker's own, without indicating whether other speakers present do or should have the same perspective. In this way she softens her claim. In the pause she puts her napkin on the table and swallows, and then delivers a remedy ‘need to have full day:, of getting you drinking’ and the diagnostic explanation ‘cause I think you're probly a bit (1.2) co:nstipay::ted.’ Again the explanation includes the word ‘think’ and ‘probably,’ which hedges the certainty with which Mum makes her claim, possibly as a way of softening a concerning explanation or an objectionable course of action.

The nature of Lanie's experience has evolved, firstly in Lanie's initial report in which she claims primary rights to report on her sensation, and secondly in Mum's response which treats the sensation as genuine and makes (mediated) claims to diagnose the nature of the experience and propose a possible remedy. In the third position space that follows in lines 13 and 14, Lanie, after another delay, responds with disagreement, saying ‘I ar:m't’, which seems to be doing something along the lines of ‘I am not’. In this way, Lanie disagrees with Mum's explanation, reporting the absence of Mum's proposed condition and by doing so re-asserts her privileged rights to access her own state. Lanie, the first pair part speaker, is here in third position, resisting the second pair part by asserting her competence. She is not aligning as an advice-recipient.

Mum then produces a form of increment to her previous turn and handles Lanie's resistance, ‘Mm:, either that or you've got a tummy bug.’ and continues her inference by adding a possible alternative. This reopens the second pair part, this time with no markers mediating her epistemic claim. Lanie, again in third position, rejects the second pair part in line 19 with ‘I've got nothing’, this time opening up her turn to address Dad in addition to Mum when she says ‘you two’.

This sequence demonstrates that both parties claim opportunities to resist or defend their or the other party's formulation in third position. Lanie rejects Mum's explanation by appealing again to her own primary access to the experience. While the rejection is taken seriously enough for Mum to produce further second pair part to defend her explanation, it does not appear to be successful in terms of discrediting Mum's account: in the subsequent talk (not shown here) Mum produces further description of Lanie's stomach condition as serious (not ‘quite right’) over a recent time period.

Example 4: Child and parent negotiate the diagnostic explanation

In this final example, Haydn asserts that his tummy is feeling different, and over several turns he works to distinguish between being sick, ill and needing the toilet. During this sequence there is a conflict in the diagnoses produced by Haydn and his mum. The sequence demonstrates how rights to report on the sensation, and the nature of that sensation, are negotiated over several turns in a more complex sequence. This extract is 3 minutes into the recording in which the serving of food and drink has been discussed. There is then a lapse in talk where this extract picks up:

Extract 4: Jephcott 6: 3.00 My tummy's feeling different

**Table:** 

04 Hay:	My tummy's feelin' bit diff'rent.
05	(.)
06 Mum:	Bit what sorry? ((Isabelle stands up on her chair))
07	(.)
08 Hay:	Different.
09 Mum:	Different. <Sit down plea:se Isa[belle. ]
10 Hay:	[I'm not ] sure
11	if I'm s:ick or not.
12	(0.6)
13 Hay:	N[ot sure if I'm s- ]
14 Mum:	[I think if you were sick] you
15	wouldn't be eating.
16	(1.4)
17 Hay:	I'm not sure if I'm sick or i::ll:.
18	(6.0) ((Children are eating. Mum is serving food.))
19 Hay:	I'm not sure if I'm sick or il[l. ]
20 Mum:	[( ] ) ring
21	me.
22	(0.1)
23 Dad:	Yeah

Haydn declares on line 4 that his tummy is feeling a bit different, as in the previous examples, claiming unmediated access to his sensation. Mum responds on line 6 by producing an other-initiated repair, for which Haydn provides the repair solution in line 8, acknowledged by Mum in line 9. She then issues a directive telling Isabelle to sit down, turning her body away from the children and towards the kitchen area of the open plan room (not marked on the transcript). Haydn develops his first pair part in lines 10–11 to propose (cautiously) that he is unsure as to whether the ‘different’ feeling indicates that he is sick. Haydn uses terms which mediate the certainty with which he asserts a diagnostic explanation for the sensation (in contrast to simply reporting the sensation itself) prefacing his turn with ‘I'm not sure’.

Following 0.6 seconds of silence Haydn begins to re-issue this turn on line 13 as Mum produces a response, asserting that if Haydn were sick he wouldn't be eating, mitigating her epistemic access with ‘I think’ which indicates that her assessment displays her own perspective rather than her experiential access. Mum is resisting Haydn's diagnostic explanation that he might be sick or ill, and she indexes observable information (Haydn eating) to strengthen the epistemic position of her disaffiliative claim. Mum resists Haydn's proposed diagnostic explanation of his sensation as being ill, on the basis that he wouldn't be eating if he was ill. Contesting Haydn's claim is oriented to as potentially problematic with the provision of evidence to support the assertion.

Haydn reasserts his proposed diagnostic explanation, reworking it to contain the options ‘sick or ill’ rather than ‘sick or not,’ presenting the possible diagnoses of tummy trouble or a more general illness. He designs his turn to more strongly prefer a response that suggests there is something the matter. After 6 seconds during which Haydn continues to eat and Mum does not respond, Haydn reissues his turn, again indicating that the response (or in this case, the silence) is deemed to be in some way deficient to the speaker's project (Antaki *et al*. 2007). Before Haydn completes his identical repeat in line 19 Mum launches something new which is partially inaudible but Dad responds to it.

In the talk that follows (shown in extract 5 below) Haydn goes on to reissue his puzzle, with a term of address, selecting Mum as recipient and pursuing a response (Stivers and Rossano 2010).

Extract 5: Jephcott 6: 3.00 My tummy's feeling different

**Table:** 

24	(0.5)
25 Dad:	˚( [ )˚ ]
26 Hay:	[I'm not sure if] I'm sick or i:ll
27	mummy.
28	(2.0)
29 Hay:	I'm probably s- I'uh probably am I'm probably
30	i:ll cause it fee:ls like it.
31	(0.2)
32 Mum:	You >wou'n't< ea:t anything if you're i:ll.
33	(1.4)
34 Hay:	[( )]
35 Isa:	[HE:::Y ( )][( )]=
36 Mum:	[( )]
37 Isa:	=di[dn't ] [( ])=
38 Hay:	[I:( ][ )]
39 Dad:	[No.]
40 Hay:	[( )]
41 Dad:	[( )]( ) today.
42 Mum:	Oh right.
43 Dad:	Where [d'you want the gravy.]
44 Hay:	[ I need a poo ] tha:t's why
45	[.h I didn't notice ]
46 Mum:	[Go and have a poo. Out!]
47 Hay:	I didn't notice I needed a poo but now I
48	no:[tice.]
49 Isa:	[M:m. ]
50 Mum:	Right.

At this point reissuing the first pair part has proved relatively unsuccessful. Haydn, still not receiving uptake from the re-issued first pair part with the additional turn-end term of address on lines 26 and 27, produces a slightly different turn on line 29 following a gap. With some difficulty in delivery (several self-repairs) he says he is probably ill (retaining uncertainty) but this time indexing his access to his own feelings with an account ‘cause it fee:ls like it..’ This first pair part contains further evidence of his account based on the notion that he has primary access to the experience. This new assertion has more certainty than his first report (upgrading from ‘not sure’ to ‘probably’) and retains a single descriptor of his state (‘ill’) rather than proposing a second alternative candidate (‘or sick’).

This assertion is successful in gaining a response. Mum produces another second pair part in line 32, maintaining her position that Haydn is not ill. However this time she formulates it in more certain terms dropping the ‘I think’ and again indexes her access to more expert knowledge of evidence of sickness and draws on the observable information about Haydn's eating. Haydn's new formulation asserting that he is probably ill, appealing to his privileged access to his experience, is rejected by Mum.

A number of turns (lines 33–42) follow which are difficult to hear. It seems that Mum and Dad begin to engage in a topic unrelated to Haydn's announced experience. Then on line 44 Haydn again issues talk related to his experience, this time announcing that he needs ‘a poo,’ and produces it as a solution and explanation for his sensation by saying ‘that's why’. This causal account for his tummy sensation is immediately resolvable, in contrast to his initial candidates of ‘sick or ill’ which are illness accounts potentially indicating a problem. It also contains no markers of mediated epistemic access or uncertainty. The delivery of the turn in overlap with Dad's turn on line 43 seems to orient to the contrast of his new interpretation as being different from anything he has previously suggested, indicating a suddenness to the realisation. After a short in-breath he produces another TCU ‘I didn't notice’ to account for why he (the owner of his experience) hadn't produced this explanation before.

In overlap with this TCU Mum responds in line 46 by directing Haydn to go to the toilet to alleviate the problem he has announced. In this way Mum accepts the explanation Haydn has proposed and the claims he makes to be able to diagnose his own experience. In line 47 Haydn reissues his explanation which was originally delivered in overlap, formulating his turn in a way that particularly emphasises the contrastive newness of his realisation that needing a poo was the cause of his tummy sensation (‘I didn't notice…but now I no:tice’). Mum issues a receipt in line 50, acknowledging this diagnosis. In this way she explicitly aligns to and agrees with his new diagnosis. Unlike her responses (and lack of responses) to Haydn's previous ‘sick or ill’ account, Mum produces her agreement to this explanation non-problematically and with no delay. Within this sequence Haydn reissues his proposed diagnostic explanation in a way that rejects his Mum's ‘not ill’ diagnosis; however this is relatively unsuccessful in eliciting an alternative response from Mum, even when he emphasises the primacy of his access to the experience. Eventually Haydn produces an alternative explanation which is immediately accepted.

Each of the five examples discussed in this analysis have demonstrated ways in which children produce expressions of sensation which claim unmediated access to their own experience, and described how an adult's response makes claims about the nature of that experience, in terms of its authenticity, severity and possible explanations and remedies. A response may formulate the sensation as serious, easily resolvable or as exaggerated, and in the subsequent interaction children display rights to accept or resist these claims.

## Discussion

By subjecting examples of children's expressions of bodily sensation in everyday family interaction to fine-grained analysis, I have pointed out the way in which children's expressions claim unmediated access to their experience and rights to report on their experience. In addition, the analysis demonstrates the negotiated character of physical sensations in interaction in terms of how they are shaped by contributions from both children and parents, and the way in which the authenticity and nature of a child's experience is produced, amended, resisted or accepted in the turns that follow, with varying degrees of success.

These findings provide clear evidence in support of the new sociology of childhood (Moran-Ellis 2010), demonstrating that children are active agents in constructing the nature of their experience. The analysis builds on this theory by showing that, specifically in regards to their health, children's pain and bodily sensation is something they themselves construct in talk. It is produced as an experience to which they maintain primary access. Yet, issuing these reports within the context of everyday family life, the child's experience is available to the recipients (parents) to be reformulated and reworked in subsequent turns.

By adopting a CA approach to everyday talk-in-interaction, this analysis has shown how the child is capable of producing descriptions of a sensation (such as a hurting tummy), and utilising resources by which to mediate (or not) their claims to access the experience. Further, an examination of the more extended sequences shows that while parents may rework the nature of this sensation in their responses, children display rights to deny or accept these formulations in the subsequent talk. In this way the analysis illustrates children's agency in terms of how they actively construct and negotiate the nature of their pain and physical experiences within family interaction. The child is not passively subject to their parents’ versions of their illness experience, in a way that would support a traditional sociological framework which treats the child as victim of external influences. Rather, within the fabric of talk between parents and children about episodes of pain, the social study of childhood's notion that children assert agency and actively contribute to the construction of their experience is exemplified. Children not only produce assertions about their physical sensations; they can, and do, object to and rework parental descriptions and causal explanations of their experience. Intergenerational relationships have already been identified as a key site in which to observe agency in the sociology of children's health (Prout and James 1997, Mayall 2001a). What this study adds is a thorough account of each turn as produced by children and adults, demonstrating the invaluable means by which CA can be used to describe, in concrete terms, the way in which intergenerational relationships between adults and children are produced and managed.

At the outset I indicated that the theoretical shift in understanding children as social actors has also been reflected in policy that promotes listening to children's views in institutional settings. Ordinary conversation has the potential to form the basis for comparative studies of institutional talk (Drew and Heritage 1992), and the patterns of mundane talk about the body and being unwell within families described in this study are relevant to the way in which children and adults interact in medical settings. For example, episodes in which children disclose a bodily sensation may precipitate entry into the medical setting. The findings in this study potentially represent the first occasion in which children can exert agency in medical decision-making. While this was not explicitly topicalised in the instances in this corpus, it is possible to see how issues to do with severity, authenticity and legitimacy become alive in these episodes. The description of resources (such as re-asserting a description or disagreeing with the other speaker's claim) by which children and parents negotiate these issues provides a foundation for future work that could examine interactions in which seeking medical help is discussed in everyday settings.

The findings in this study are also relevant to medical settings in terms of identifying similarities with patterns of talk presented in this article, and those described in existing studies of talk in institutional environments. For example, the current study describes the resources which children and parents use to make epistemic claims and exert agency in relation to the nature of their experience. These resources include the way in which children claim unmediated access to an experience by producing descriptions with no epistemic markers, or assert rights to confirm or resist a parent's description. Comparatively previous research has shown that adult patients (who like the children in the current study are the ‘experiencers’) construct themselves as having superior knowledge of their illness experience (Heritage 2006, Heritage and Robinson 2006, Peräkylä 1998). Further work is needed to examine if and how the resources by which to handle these sorts of issues, developed in everyday talk, are available to be used by adults and children in institutional settings.

In conclusion, a CA approach represents an effective methodological technique for exploring topics within the framework of a new sociology of childhood. By closely examining what children and adults say, and how they say it, a CA study is able to bypass many of the problematic power relations inherent in alternative forms of research that ask children to describe or reflect on such interactions. A CA study is ideal for facilitating a detailed description of the child's role as a social actor, and the child's everyday participation in their health care.

## Notes

1 Thanks to Sarah Crouch and Alexandra Kent for permission to use these data.

2 The expressions of bodily sensation in this study, while projecting a response (and therefore representing an initiating action), also display that there was a ‘source’ that preceded it (see Schegloff's 2007 description of retro-sequences). This example, unlike the others, appears more explicitly to contain a clear cause of the child's bodily expression – the sound of the scraping saucepan. In other sequences the cause of the sensation is less clear. The expressions themselves are what surface as the first effective component in the interaction, signalling that the sequence is in progress. While it is interesting to note that in this case the source is more explicit, any analytic distinction between sensations with external or internal causes was analysed as a participants’ concern, as part of the ongoing issue of the degree to which information about the nature and severity of the expressed sensation is revealed.
